# Evaluation of Depth Size Based on Layered Magnetization by Double-Sided Scanning for Internal Defects

**DOI:** 10.3390/s24113689

**Published:** 2024-06-06

**Authors:** Zhiyang Deng, Dingkun Qian, Haifei Hong, Xiaochun Song, Yihua Kang

**Affiliations:** 1 Key Lab of Modern Manufacture Quality Engineering, Hubei University of Technology, Wuhan 430068, China; 2 Huazhong University of Science and Technology, Wuhan 430074, China; yihuakang@hust.edu.cn

**Keywords:** stepped magnetization, magnetic permeability perturbation, defect size evaluation, ferromagnetic material

## Abstract

The quantitative evaluation of defects is extremely important, as it can avoid harm caused by underevaluation or losses caused by overestimation, especially for internal defects. The magnetic permeability perturbation testing (MPPT) method performs well for thick-walled steel pipes, but the burial depth of the defect is difficult to access directly from a single time-domain signal, which is not conducive to the evaluation of defects. In this paper, the phenomenon of layering of magnetization that occurs in ferromagnetic materials under an unsaturated magnetizing field is described. Different magnetization depths are achieved by applying step magnetization. The relationship curves between the magnetization characteristic currents and the magnetization depths are established by finite element simulations. The spatial properties of each layering can be detected by different magnetization layering. The upper and back boundaries of the defect are then localized by a double-sided scan to finally arrive at the depth size of the defect. Defects with depth size of 2 mm are evaluated experimentally. The maximum relative error is 5%.

## 1. Introduction

Ferromagnetic components such as nuclear power second-loop pipeline boilers are usually operated under extreme environments such as high temperature and high pressure for a long period of time and are very prone to corrosion, cracks, and other internal damage [1,2,3]. From a practical production point of view, an overestimation of the hazards of the defective size would lead to unnecessary stoppages for repairs and significant economic losses. By accurately evaluating the depth size of the defect, we can more accurately determine the effect of the defect on the overall properties of the material. This will help us to decide whether there is a need to stop the pile for repairs as well as the scope and extent of the repairs. Therefore, the accurate detection of internal defects and obtaining critical size information are extremely important.

In the current research, the common electromagnetic nondestructive evaluation methods for defect size evaluation include eddy current testing (ECT) [4,5,6,7], pulsed eddy current testing (PECT) [8,9,10,11,12], and magnetic flux leakage (MFL) testing [13,14,15], alternating current field measurement (ACFM) [16,17,18]. MFL [19,20,21,22,23] was limited by wall thickness, while the signal for evaluating defects in thick-walled components is attenuated with increasing wall thickness, and some of them [24,25,26,27,28,29,30] were often affected by skin effects or magnetic shielding effects. Nowadays, some scholars [31,32,33,34] evaluated defects through signal characterization with algorithmic optimization. Several academics investigated [6,15,35,36] making defect evaluation possible by optimizing sensors such as array probes. These required extensive experimental data analysis and mathematical modeling iteration. Some scholars [7,14,19,23,37] evaluated defects by detecting principle innovations, such as composite magnetic fields, propagation compensation factor (PCF), etc. The aforementioned evaluation methods facilitate the reduction in data processing at a subsequent stage.

It was widely accepted by scholars that when magnetization is applied to a ferromagnetic material, the density distribution of magnetic lines in the same cross-section of a ferromagnetic member is uniform if there were no internal defects and no structural mutations [38,39,40]. Furthermore, some scholars [41,42] also conducted research into inhomogeneous magnetic fields. They achieved uniform magnetization in the horizontal direction of the detection interval by changing the angle between the magnetic poles but did not take the depth direction into consideration. In the previous study, the magnetic permeability perturbation testing (MPPT) method based on the nonlinear magnetization properties of ferromagnetic materials was proposed to detect internal defects and surface defects in thick-walled steel pipes [43,44], but it cannot directly obtain the defect size information from the time-domain signal [45]. Based on the non-uniform uneven penetration property of the magnetic field, the magnetized layering is achieved by applying a magnetic field of varying intensity. Layering information can determine the upper and lower boundaries of the defect. In the case, the depth size information of the defects can be obtained by double-sided scanning. The double-sided scanning allows for obtaining more layers of information than other methods, which makes it easier to evaluate the depth size of the defects.

The main structure is as follows: Section 2 analyzes the relationship between the magnetization strength and the magnetization depth. Section 3 investigates the relationship between the magnetization strength, magnetization depth, and defect depth size by establishing a finite element model. Section 4 builds an experimental platform to verify the effectiveness of the proposed method. Section 5 discusses the effect of defect width on the peak value of the detected signal. Finally, brief conclusions are given in Section 6.

## 2. Layered Magnetization Mechanism

**Figure 1:**
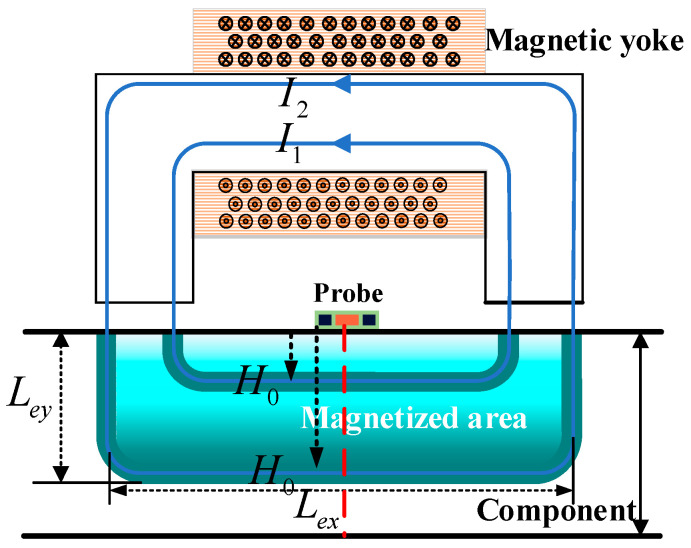
Magnetization model.

In this section, the magnetization model of a U-shaped magnetizer with a thick-walled member is established as shown in Figure 1.

Since the magnetic field excited by the U-shaped magnetizer is non-uniform, especially near the magnetic poles, this non-uniformity is more prominent, and thus the magnetization of the material is also non-uniform. To calculate the magnetic field distribution inside the material, it is first necessary to calculate the magnetic field distribution of a U-shaped magnetizer in a vacuum. The magnetic field strength Equation (1) can be obtained from the Ampere loop theorem:

(1) $$\oint_{l}Hdl=NI$$

In Equation (1), N is the number of turns, I is the actual current value, and l is the effective magnetic circuit length.

In the magnetizing field of a U-shaped magnetizer, the magnetic field strength $\vec{H}$ at different points is calculated by the formula:

(2) $$\vec{H}=\frac{NI}{L_{ex}}\vec{x}+\frac{NI}{L_{ey}}\vec{y}$$

In Equation (2), $\vec{H}$ is the magnetic field intensity, N is the number of turns, I is the actual current value, $L_{ex}$ is the effective magnetic circuit length in the horizontal direction, and $L_{ey}$ is the effective magnetic circuit length in the longitudinal direction. $\vec{x}$ is a unit vector in the transverse direction in 2D space, and $\vec{y}$ is the unit vector in the vertical direction in 2D space. In this article, the symbols $\vec{x}$ and $\vec{y}$ are solely utilized to denote directionality, which is devoid of any inherent physical significance or quantitative representation.

Assuming that the magnetic field intensity decays to $H_{0}$, $H_{0}$ is an infinitesimal value, and the point at this point is identified as the limit point of the magnetic field. When the magnetized point is on the centerline of the magnetizer (i.e., when the transverse effective length $L_{ex}$ is unchanged, see the red line in Figure 1), the decay distance required of $L_{ey}$ increases as the magnetizing current increases and the magnetic field strength decays to $H_{0}$.

**Figure 2:**
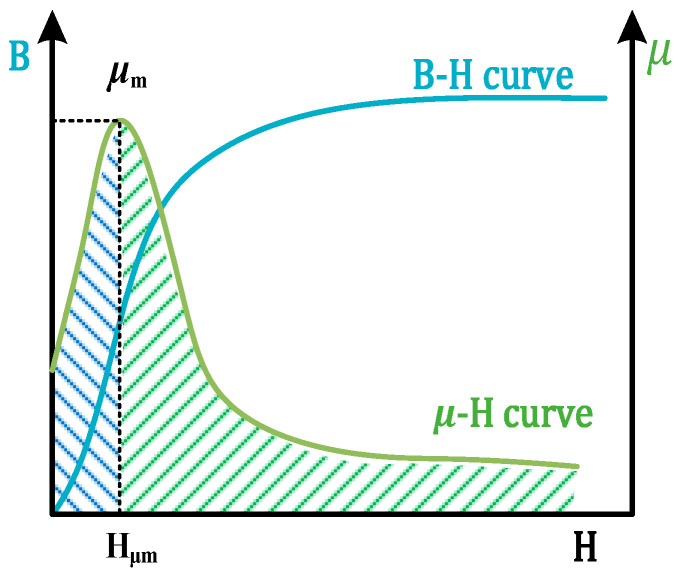
Ferromagnetic member B−H and μ−H curves.

In ferromagnetic material, the magnetic field increases with the applied DC magnetizing field H. The magnetic permeability gradually increases to a maximum value and then shows a decreasing trend, as shown in Figure 2.

The relationship between magnetic field strength and magnetic induction is expressed as

(3) $$B=\mu_{0}\mu_{r}H=\mu H$$

In Equation (3), μ is the magnetic permeability of the component, $\mu_{0}$ is the vacuum permeability, and $\mu_{r}$ is the relative magnetic permeability. In Figure 2, $\mu_{m}$ is the maximum value of magnetic permeability, and $H_{\mu_{m}}$ is the magnetic field intensity at $\mu_{m}$.

**Figure 3:**
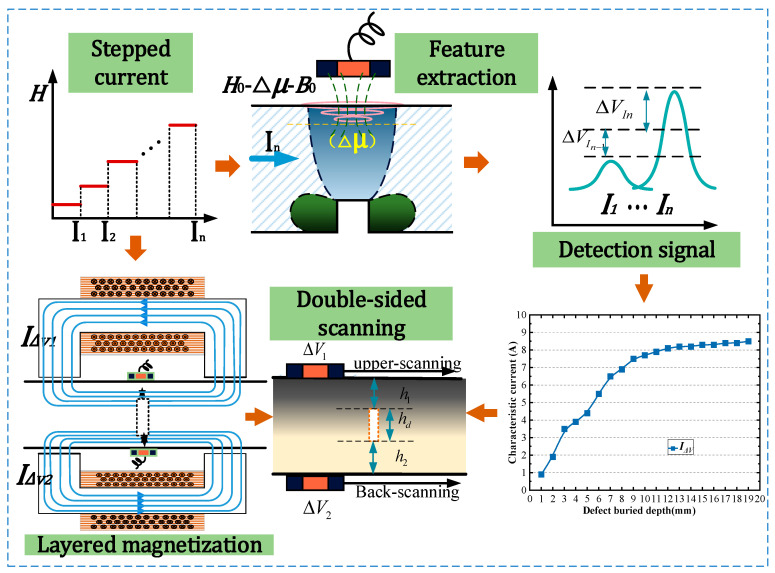
Schematic diagram of layered magnetization evaluation principle.

The static magnetizing field is excited by a DC magnetizer, and a step change in the magnetic field is achieved by increasing the magnitude of the DC current. Under the action of the step magnetic field, the magnetic field lines are disturbed by defects, producing permeability perturbations. These perturbations generate detection signals through the eddy current field and secondary magnetic field changes. By subtracting the peak values of the signals under magnetizing currents of stepped intensities, the signal increment $\Delta V_{I_{n}}=V_{I_{n}}-V_{I_{n-1}}$ is obtained. The signal amplitude increment increases with the magnetizing current. The maximum $\Delta V_{I_{n}}$ occurs when the magnetizing current causes the magnetization depth to coincide with the defect upper boundary. Subsequently, the magnetizing current increases as the signal amplitude increment decreases. We recorded the current corresponding to the maximum value of each signal increment as the “characteristic current” $I_{\Delta V}$. For defects with different burial depths, the $I_{\Delta V}$ is different. The localization of defects can be achieved by establishing a relationship between $I_{\Delta V}$ and defect burial depth h. By applying double-sided step magnetization with double-sided scanning, it is possible to determine the characteristic current is $I_{\Delta V1}$ when the defect is swept from the upper surface and the characteristic current is $I_{\Delta V2}$ when the defect is swept from the back surface. Then, the spatial information of each magnetization layering above and below can be determined. The distance $h_{1}$ to the upper surface of the defect is obtained by substituting the value $I_{\Delta V1}$ into the characteristic current curve, and the distance $h_{2}$ to the under surface of the defect is obtained by substituting the value $I_{\Delta V2}$ into the characteristic current curve. The depth size $h_{d}$ of the defect is obtained by subtracting the distance $h_{1}$ and $h_{2}$ from the wall thickness (Figure 3).

## 3. Simulation

### 3.1. Three-Dimensional (3D) FEM Model

**Figure 4:**
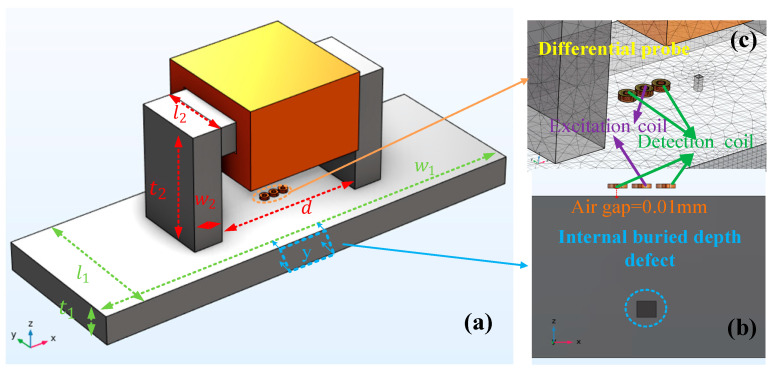
Simulation model.

In this section, a finite element model of the MPPT method is built by COMSOL Multiphysics 5.6, as shown in Figure 4. Figure 4a shows the schematic diagram of the sizes of the magnetizer and the specimen, Figure 4b shows the internal defect of the specimen, and Figure 4c shows the differential probe. The detection probe contains an excitation coil and two detection coils. The excitation coil is in the center and the detection coils are on both sides, and the spacing between coils is 0.1 mm. The analysis explores the distribution of magnetic permeability perturbation (MPP) inside the material under step magnetization. A set of defects with different burial depths is detected by the probe, and the signals are used to corroborate the relationship between the magnetizing current and the magnetization depth of the measured steel plate. The specific parameters in the simulation are shown in Table 1. The Figure 4a presents an overall schematic of the simulation model, Figure 4b shows the detailed view of the probes and the defect, and Figure 4c depicts a cross-sectional view y of the defect portion in the xoz plane.

This model is solved in two stages. In the first step, the magnetization field distribution in the workpiece is obtained by a steady-state solver. The variable intensity DC magnetization is generated by adjusting the magnetizing current. In the second step, the results of the steady-state solver are called in the frequency domain to calculate the relationship between the permeability and the output of the probe. The material properties of the U-shaped yoke and the steel plate are set to No. 45 steel, which is shown in Table 2.

### 3.2. Magnetization Depth of Different Magnetizing Current

**Figure 5:**
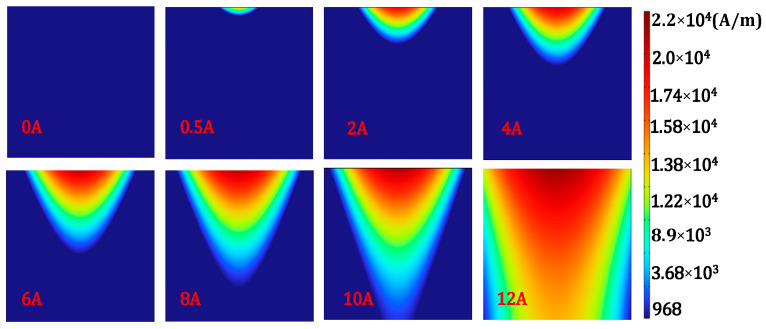
Effect of magnetizing current on magnetization depth.

When there are no defects in the steel plate, the magnetization cloud map of the local region of the xoz chapter(20 mm × 20 mm) at the center of the steel plate is extracted, as shown in Figure 5. The internal magnetic field of the ferromagnetic component changes unevenly with the increase in the applied magnetic field strength. The magnetic field penetrates downwards from the surface, and the magnetic field strength gradually decreases, but the range of magnetization inside the steel plate increases along the depth direction.

**Figure 6:**
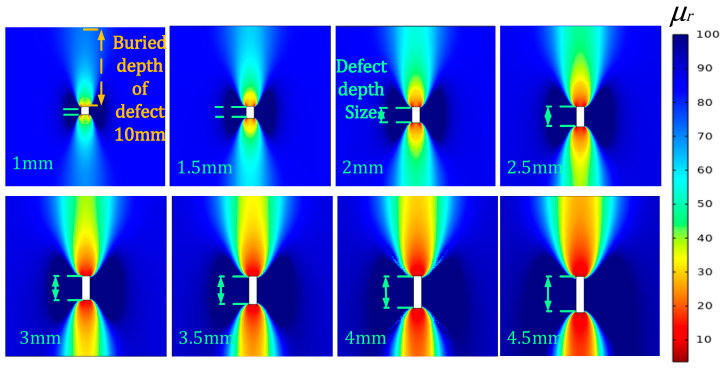
Perturbation graph of magnetic permeability for defects of different depth sizes.

From Figure 6, under the influence of magnetization, the perturbation of the magnetic permeability of the defects inside the component spreads not only to the upper surface but likewise to the back surface. Because the upper and the lower boundaries of the defects are at different distances from the surface, the values of permeability perturbation signals are different.

**Figure 7:**
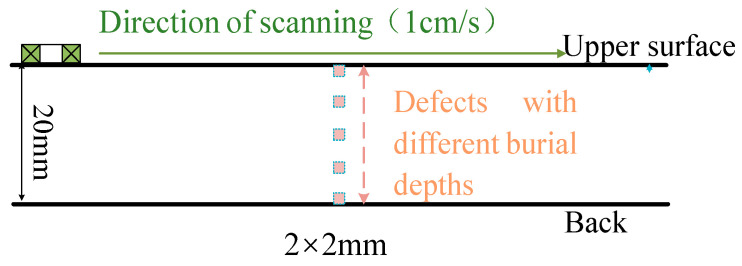
Extraction path.

The MPP of the surface layer is considered due to the skin effect. For defects with different burial depths, the differential probe signals on the surface of the specimen are extracted, and the extraction path is shown in Figure 7. For each burial depth, a magnetic field with a step change in intensity is applied to the defects. The MPP at each magnetizing current is extracted.

**Figure 8:**
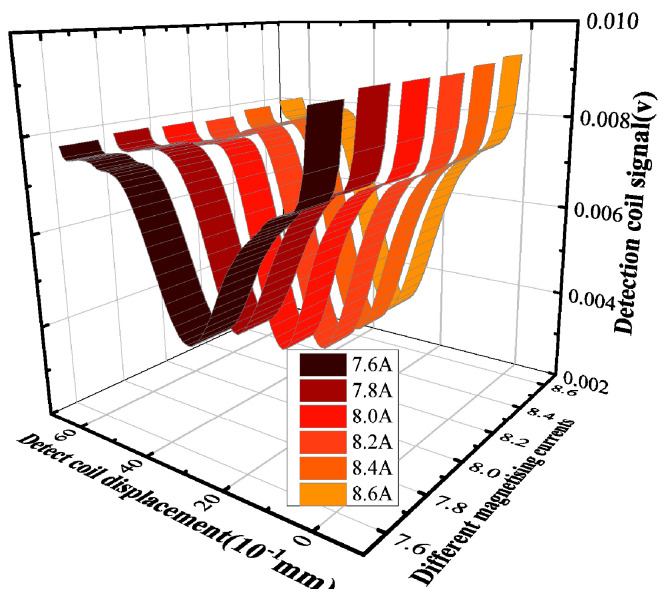
Signals of defects buried at 10 mm depth under different magnetizing currents.

**Figure 9:**
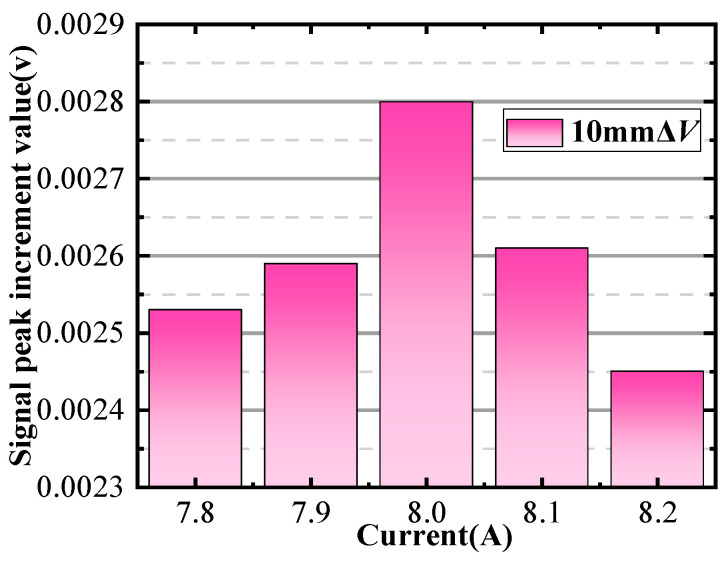
The relationship between signal peak and magnetization current.

In the simulation process, the magnetizer is subjected to a current that varies in steps of 0.1 A. The lift-off distance of the differential probe is 0.05 mm, and the defect depth is 10 mm. Due to the large amount of data, the following display is part of the results that can show the relationship of $\Delta V_{I_{n}}$ between $I_{\Delta v}$. The detection signals at magnetizing currents of 7.6 to 8.6 A are obtained by the scanning method in Figure 7, as shown in Figure 8. Based on the method described in Section 2, the signal amplitude increment $\Delta V_{I_{n}}$ is extracted as shown in Figure 9. The peak increment $\Delta V_{I_{n}}$ of the detected signal firstly increases with the increase in the current and starts to decrease when the magnetizing current reaches 8 A. Therefore, 8 A is the characteristic current corresponding to the maximum value of the $\Delta V_{I_{n}}$. Because when the magnetization reaches exactly the upper boundary of the defect, the permeability perturbation signal change is from metal permeability to air permeability. Consequently, any further increase in the signal can be attributed to a growing proportion of air within the defect, resulting in a relatively smaller incremental change.

**Figure 10:**
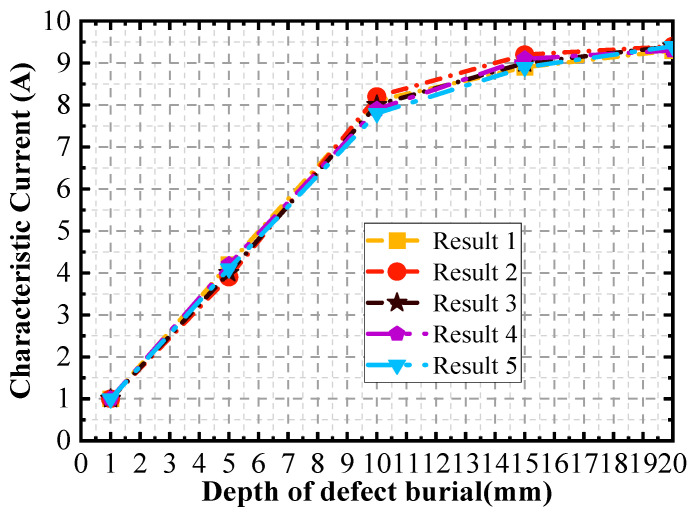
Curve of characteristic current versus depth of defect burial.

Further, the burial depth of the defects is varied, and the defects are set up sequentially from the surface to the bottom at intervals of 1 mm. A set of signals at different currents for each defect with different burial depths is extracted. After five simulation experiments, the relationship between the characteristic currents $I_{\Delta v}$ and the magnetization depth of the signal is shown in Figure 10. The maximum relative error of the curves of 3% occurs near the burial depths of 11 and 12 mm.

### 3.3. Measurement Signal and Error Analysis

**Figure 11:**
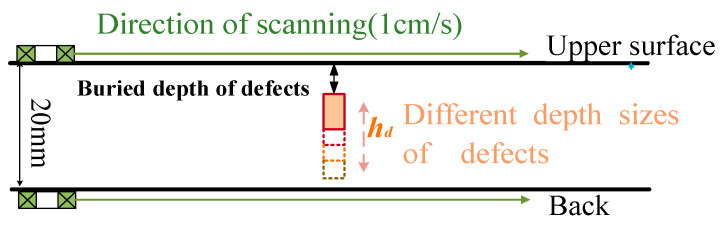
Double-sided scanning method.

The detection methods for double-sided scanning are specified as follows (Figure 11): different layering information on the upper and back surfaces is obtained by applying double-sided step magnetization with double-sided scanning. By applying double-sided step magnetization with double-sided scanning, it is possible to determine the characteristic current $I_{\Delta V1}$ when the defect is swept from the upper surface and the characteristic current $I_{\Delta V2}$ when the defect is swept from the back surface. In turn, the spatial information of each magnetization layering above and below can be determined. The distance $h_{1}$ to the upper surface of the defect is obtained by substituting the value $I_{\Delta V1}$ into the characteristic current curve. The distance $h_{2}$ to the under surface of the defect is obtained by substituting the value $I_{\Delta V2}$ into the characteristic current curve. The depth size $h_{d}$ of the defect is obtained by subtracting the distance $h_{1}$ and $h_{2}$ from the wall thickness.

**Figure 12:**
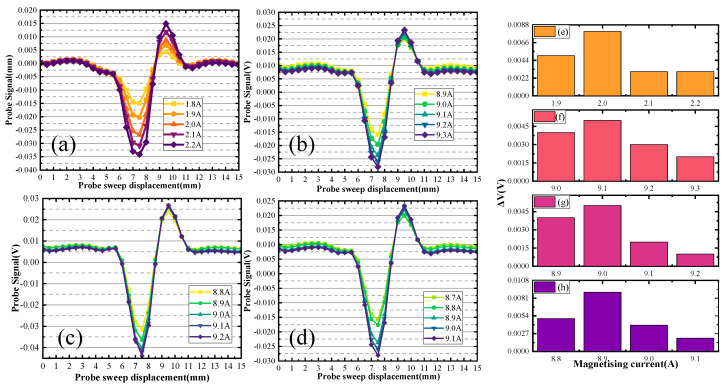
Detection signal for defects with a burial depth of 2 mm.

**Figure 13:**
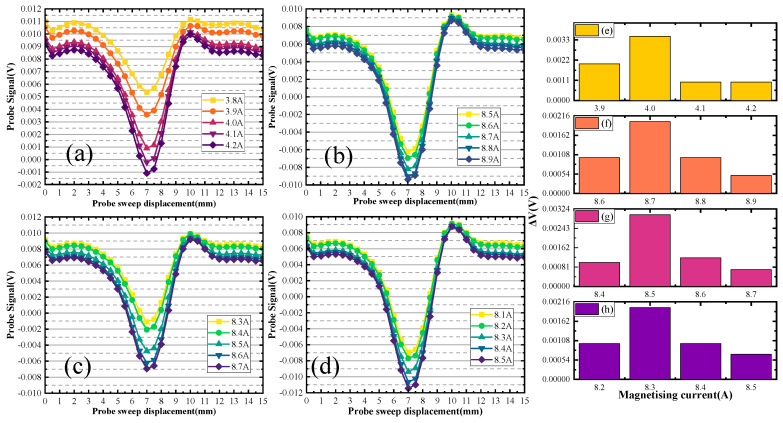
Detection signal for defects with a burial depth of 5 mm.

**Figure 14:**
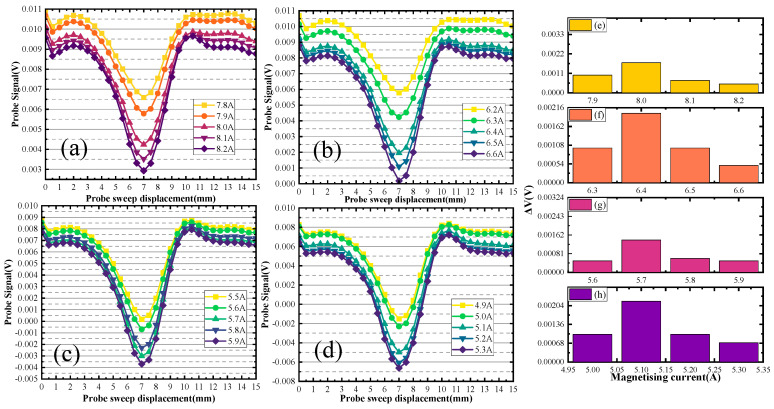
Detection signal for defects with a burial depth of 10 mm.

Different sets of defect size detection signals are plotted in Figure 12, Figure 13 and Figure 14.

When the defect depth is 2 mm, the defect scanning signals of different depth sizes are shown in Figure 12. Figure 12a,b illustrate the upper scanning characteristic current value of the first group of defects, which is 2.0 A. Substituting the characteristic current into the curve gives the distance. The distance of the defect upper boundary from the upper surface of the specimen is 2.2 mm. The characteristic current value of the back scanning is 9.1 A, and distance of the defect lower boundary from the back surface of the specimen is 15.9 mm. The defect depth size is calculated to be 1.9 mm. In Figure 12a,c, the double-sided scanning generated characteristic currents of 2 A and 9 A. The distance of the defect upper boundary from the upper surface of the specimen is 2.2 mm, and the distance of the defect lower boundary from the back surface of the specimen is 14.9 mm. The defect depth size is calculated to be 2.9 mm. In Figure 12a,d, the double-sided scanning generated characteristic currents of 2 A and 8.9 A. The distance of the defect upper boundary from the upper surface of the specimen is 2.2 mm, and the distance of the defect lower boundary from the back surface of the specimen is 14.1 mm. The defect depth size is calculated to be 3.7 mm. Figure 12e–h represents the change ΔV of the signal from Figure 12a–d.

When the defect depth is 5 mm, the defect scanning signals of different depth sizes are shown in Figure 13. In Figure 13a,b, the double-sided scanning generated characteristic currents of 4 A and 8.7 A. The distance of the defect upper boundary from the upper surface of the specimen is 5 mm and the distance of the defect lower boundary from the back surface of the specimen is 13.1 mm. The defect depth size is calculated to be 1.9 mm. In Figure 13a,c, the double-sided scanning generated characteristic currents of 4 A and 8.5 A. The distance of the defect upper boundary from the upper surface of the specimen is 5 mm, and the distance of the defect lower boundary from the back surface of the specimen is 12.1 mm. The defect depth size is calculated to be 2.9 mm. In Figure 13a,d, the double-sided scanning generated characteristic currents of 2 A and 8.3 A. The distance of the defect upper boundary from the upper surface of the specimen is 5 mm, and the distance of the defect lower boundary from the back surface of the specimen is 11 mm. The defect depth size is calculated to be 4 mm. Figure 13e–h represents the change ΔV of the signal from Figure 13a–d.

When the defect depth is 10 mm, the defect scanning signals of different depth sizes are shown in Figure 14. In Figure 14a,b, the double-sided scanning generated characteristic currents of 8 A and 6.4 A. The distance of the defect upper boundary from the upper surface of the specimen is 10 mm, and the distance of the defect lower boundary from the back surface of the specimen is 8 mm. The defect depth size is calculated to be 2 mm. In Figure 14a,c, the double-sided scanning generated characteristic currents of 8 A and 5.7 A. The distance of the defect upper boundary from the upper surface of the specimen is 10 mm, and the distance of the defect lower boundary from the back surface of the specimen is 7 mm. The defect depth size is calculated to be 3 mm. In Figure 14a,b, the double-sided scanning generated characteristic currents of 8 A and 5 A. The distance of the defect upper boundary from the upper surface of the specimen is 10 mm, and the distance of the defect lower boundary from the back surface of the specimen is 6.0 mm. The defect depth size is calculated to be 4.0 mm. Figure 14e–h represents the change ΔV of the signal from Figure 14a–d.

**Figure 15:**
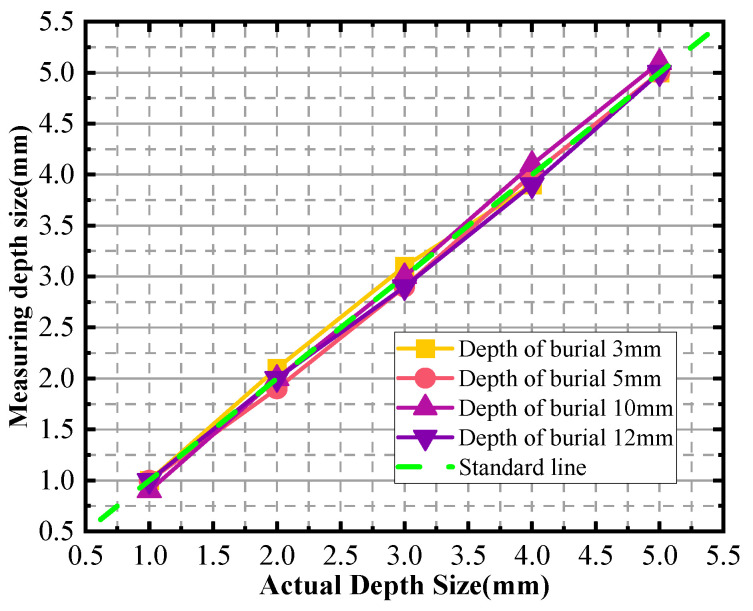
Depth size measurement charts for different burial depths.

In Figure 15, the actual sizes of the defects are represented as the horizontal coordinates, while the inspection sizes derived from the experimental measurements are plotted along the vertical axis. This graphical representation serves to analyze and visualize the potential errors in the detection process. Table 3 provides a comprehensive list of the actual sizes of the defects along with the evaluated sizes obtained at various burial depths. It also includes the relative errors, which indicate the deviation between the measured and actual sizes expressed as a percentage of the true value.

Based on the analysis of the results from the four simulation groups, the maximum relative error is calculated to be 7.5%. This figure highlights the precision and accuracy of the detection method, despite the inevitable presence of some error. The simulation results further support this conclusion. Specifically, the double-sided scanning technique is shown to be effective in determining the upper and lower boundaries of the defects based on the characteristic current curves. This approach allows for a more accurate evaluation of the defect depth sizes. The analysis of the relative error associated with this method reveals that it is also within an acceptable range with a maximum of 7.5%. This demonstrates the reliability and accuracy of the double-sided scanning technique in defect detection and sizing.

## 4. Experimental Results and Analysis

### 4.1. The Testing System and Specimen

**Figure 16:**
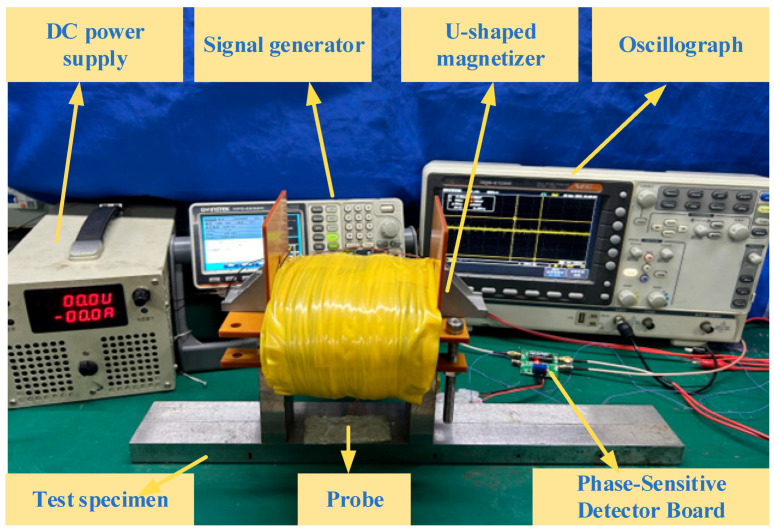
Experimental platform.

**Figure 17:**
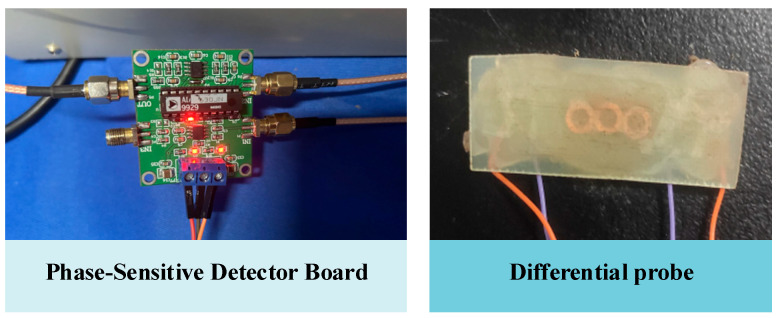
Probe and filter circuitry.

The experimental platform consists of a DC magnetization power, a signal generator, a signal amplification-conditioning module, a U-shaped magnetizer, a probe, and a specimen, as shown in Figure 16. The magnetizing coil is loaded with a DC current of the step change in intensity to produce different magnetic fields. The magnetizing coil is 989 turns with a wire diameter of 1 mm, and the yoke size is the same as the simulation model. The probe consists of three coils with an outer diameter of 4 mm, an inner diameter of 2.5 mm, and a height of 0.85 mm. The excitation coil is in the middle, and the other two coils are the receiving coils. All of them are 100 turns, and their wire diameters are 0.05 mm. The signal generator provides the excitation coil with an alternating current at a frequency of 100 kHz and a voltage of 1 V, as shown in Figure 17.

**Figure 18:**
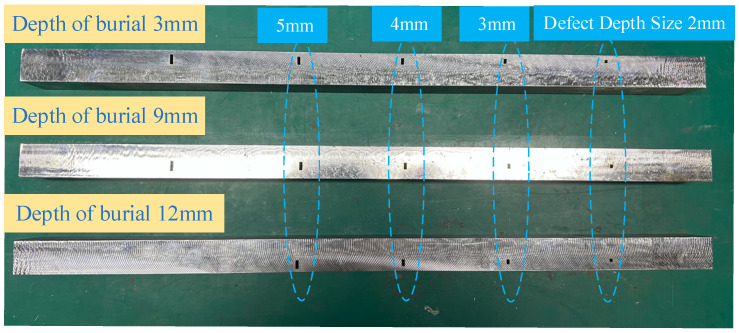
Experimental specimens.

The specimens are No. 45 steel plates with sizes of 400 mm × 100 mm × 20 mm. Blind holes of 2 mm, 3 mm, 4 mm depth lengths are machined on the side of the specimen to simulate internal defects. The burial depths of them are 3, 9, and 12 mm from the positive side of the plate, as shown in Figure 18.

For defects with different burial depths, the magnetizing current is increased in steps of 1 A from 0 to 12 A. At each current, the probe swept over the surface of the specimen at a uniform speed (the direction of scanning is shown in Figure 11). The detection signal is acquired by an oscilloscope after passing through the amplification and conditioning module.

### 4.2. Experimental Results

**Figure 19:**
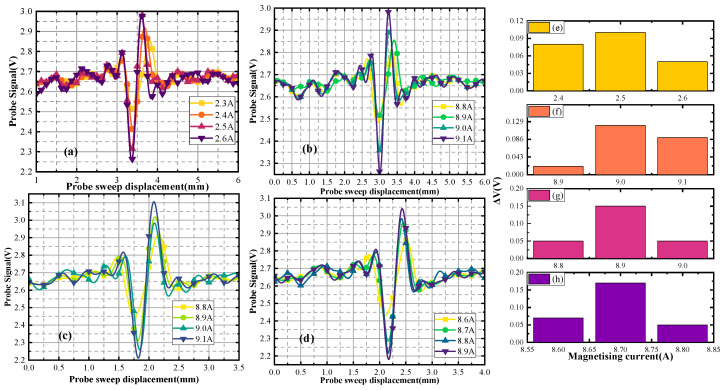
Detection signals for different depth sizes (depth of buried = 3 mm).

To replicate the method used in this paper, place the specimen on the platform, place the DC magnetizer on the upper surface of the specimen, connect the DC power supply to the DC magnetizer, and apply the step magnetizing current. The excitation coil in the probe is then connected to a sine wave signal generator, and the receiver coil is connected to the input port of the phase-sensitive detector board. The output port of the phase-sensitive detector board is connected to an oscilloscope. Scanning the upper surface of the specimen by the probe yields a defect signal scanning signal, as shown in Figure 19a. The plate is then placed in reverse, and the back of the plate is swept to reveal the defect signals shown in Figure 19b–d.

Different sets of defect size detection signals are plotted in Figure 19.

In Figure 19a, the up-sided scanning generated a characteristic current of 2.5 A. The defect distance from the up surfaces is 3.0 mm. In Figure 19b, the back-sided scanning generated a characteristic current of 9.0 A. The defect distance from the back surfaces is 14.9 mm. The defect depth size is calculated to be 2.1 mm. In Figure 19c, the back-sided scanning generated a characteristic current of 8.9 A. The defect distance from the back surfaces is 14.1 mm. The defect depth size is calculated to be 2.9 mm. Figure 19d shows that the back-sided scanning generated a characteristic current of 8.7 A. The defect distance from the back surfaces is 13.1 mm. The defect depth size is calculated to be 3.9 mm. Figure 19e–h represents the change ΔV of the signal from Figure 19a–d.

**Figure 20:**
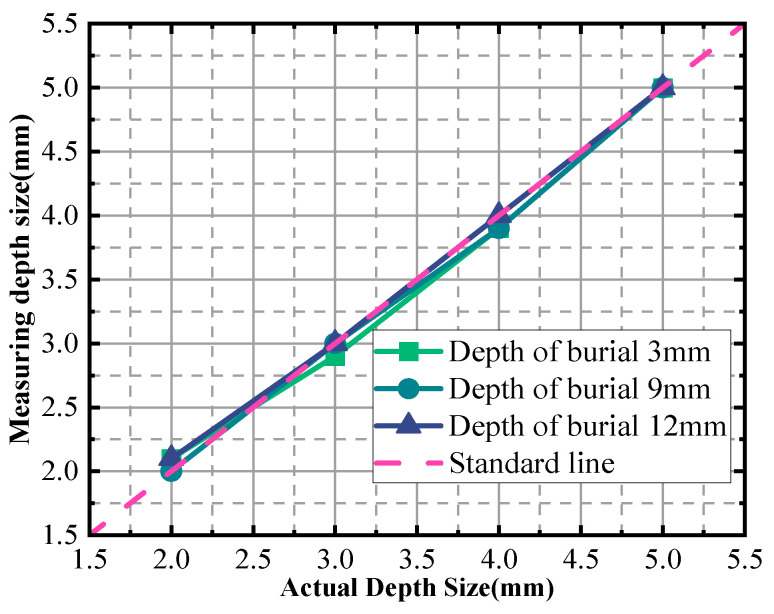
Depth size measurement charts for different burial depths.

In Figure 20, the horizontal coordinates represent the actual sizes of the defects, while the vertical coordinates depict the inspection sizes obtained through the measurement process. The purpose of this graphical representation is to analyze and visualize the errors associated with defect detection. Notably, the graph reveals a relative error of up to 5% when detecting a defect with a depth size of 2 mm.

To provide a more detailed overview, Table 4 lists the actual sizes of the defects along with the evaluated sizes at various burial depths and the corresponding relative errors. This table serves as a quantitative reference, allowing us to compare the measured sizes against the true values and calculate the accuracy of our detection method. The relative error indicates the deviation of the measured size from the actual size, which is expressed as a percentage of the actual size.

## 5. Discussion

Through simulation and experiment, we can find that the maximum relative error of this evaluation of the depth size is 7.5%. The reason for these errors may be the nonlinear variation in the characteristic current curve. In order to avoid the characteristic current curve being affected by other defect sizes such as width, this section focuses on the effect of defect width on the characteristic current curve.

**Figure 21:**
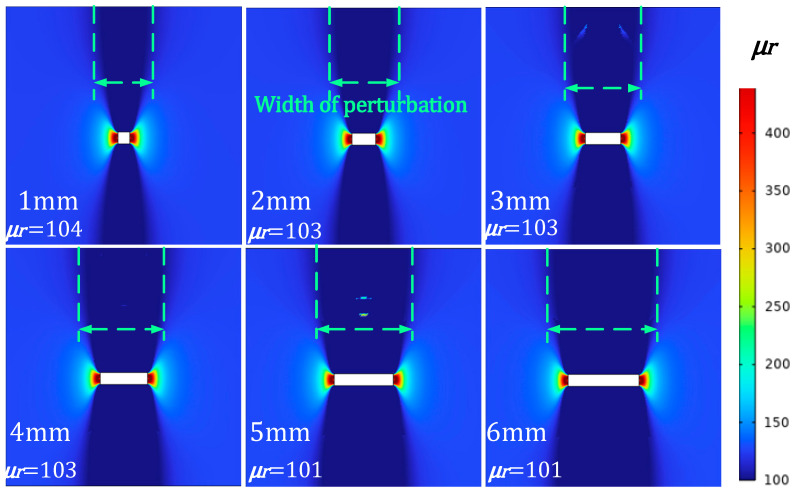
Different defect widths.

**Figure 22:**
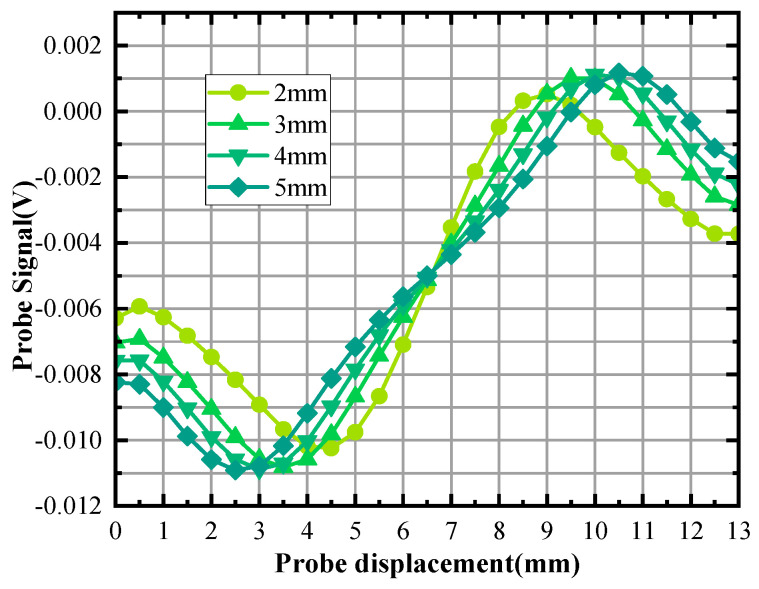
Different width detection signals.

In the actual inspection, defects may extend not only in the direction of depth but also in the direction of width. Therefore, the detection signal will not only contain information about the depth of the defect but also about the width of the defect (In Figure 21). The cloud diagram of magnetic permeability perturbation with different defect widths is shown in Figure 22.

In Figure 21, there is no discernible change in permeability perturbations as a function of defect width. The range of magnetic permeability perturbations has expanded. Figure 22 shows the scanning signal at a consistent burial depth for defects of varying widths.

As can be seen in Figure 22, the signal peaks do not increase significantly with the increasing defect width. The size of the defect width has little effect on the peak increment of the detected signal. The distance between the peaks of the defect signal increases as the defect width increases.

**Figure 23:**
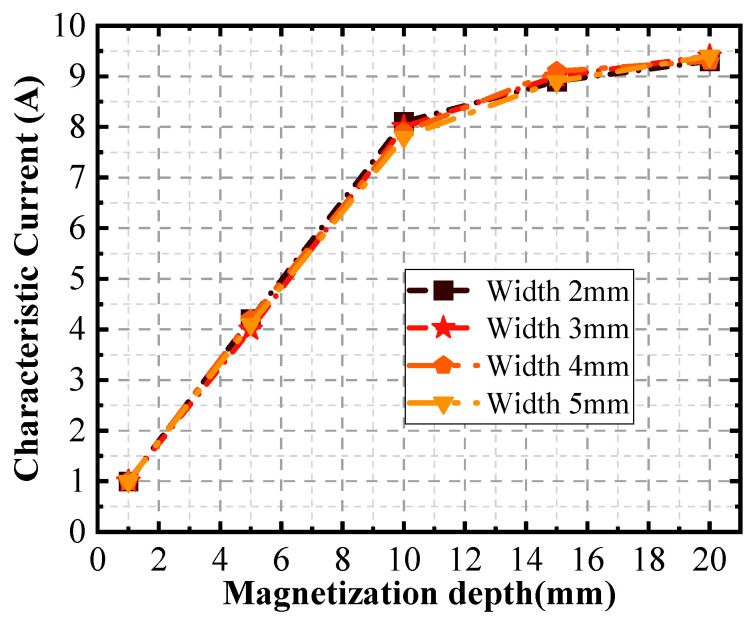
Curves of magnetization depth as a function of the characteristic current for different defect widths.

Figure 23 presents a compelling case that the width of a defect does not exert a substantial influence on the characteristic current. This observation signifies that the distance between the scanning surface and the defect can be accurately determined based solely on the analysis of the characteristic current curve. In other words, regardless of the width variation in the defect, the characteristic current remains relatively stable, making it a reliable indicator for distance measurement.

**Figure 24:**
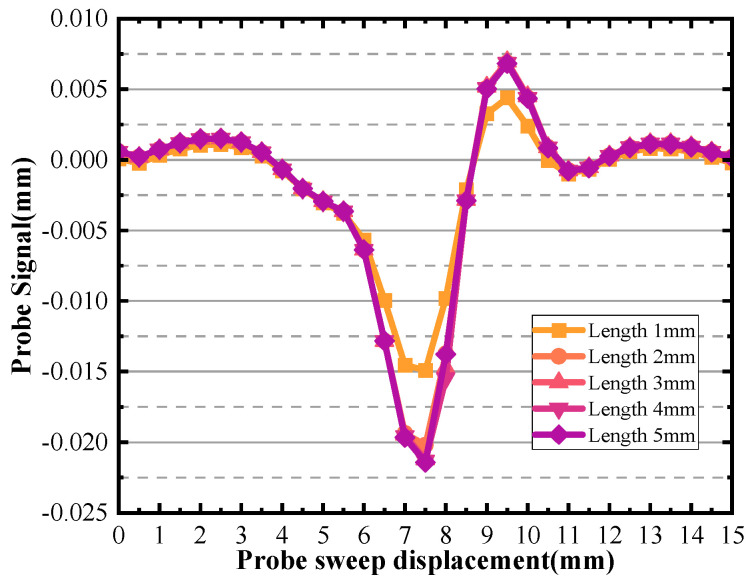
Different length detection signals.

In Figure 24, it is observed that the majority of the signal peaks are clustered closely together with a notable exception of length 1 mm. The underlying cause for this disparity in signals could be attributed to the fact that the defect length is significantly shorter than the diameter of the probe being used. This disparity leads to the detection of smaller peaks in the signal corresponding to the defective area. The probe’s diameter determines the region it can effectively sense, and if the defect is smaller than this diameter, the detected signal may not be as strong or as distinct as one would expect from a larger defect.

**Figure 25:**
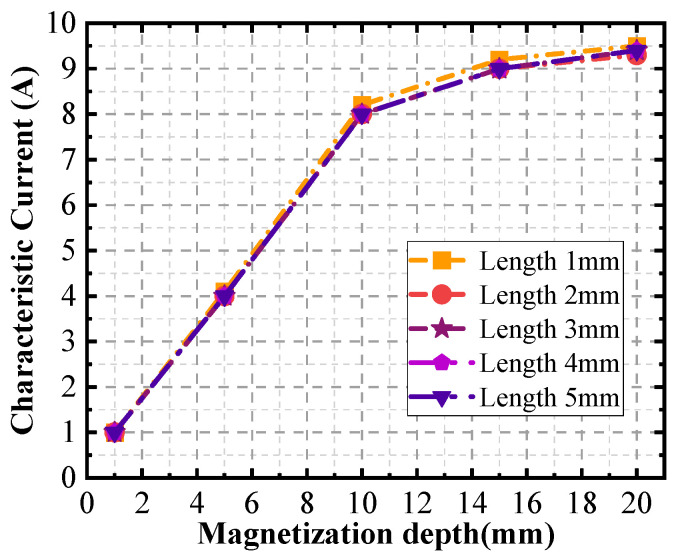
Curves of magnetization depth as a function of the characteristic current for different defect lengths.

Furthermore, the same logic applies to the length of the defect. The figure also shows that the effect on the characteristic current is not significant for defect lengths larger than the probe diameter. This indicates that when assessing material surface defects using current detection techniques, precise knowledge of the defect’s length may not be as crucial as understanding the variations in the characteristic current curve. In summary, Figure 23 and Figure 25 underscores the fact that when utilizing characteristic current measurements to detect and evaluate surface defects, the distance between the scanning surface and the defect can be accurately determined by analyzing the characteristic current curve, while the specific sizes of the defect, such as width and length, have a relatively minor impact on the current curve. This insight can significantly enhance the efficiency and accuracy of defect detection methods.

## 6. Conclusions

In this paper, a defect depth size evaluation method based on layered magnetization by double-sided scanning is proposed, which can effectively detect the defect depth size. The changes in magnetic field intensity under the excitation of different magnetizing currents are investigated. The phenomenon of internal magnetization depth stratification is revealed when the specimen is subjected to different magnetic field intensities. The characteristic current values are determined from the signal incremental change information. The relationship between the characteristic current and the magnetization depth is established. The distance between the defect boundary and the surface of the component can be effectively localized by using the characteristic current. Then, the upper and back boundary positions of the defects are determined by double-sided step magnetization with double-sided scanning.

In the simulation phase, the relationship curve between the magnetization depth and magnetization current was determined by setting defects with different embedded depths. Additionally, the simulation employed the double-sided scanning to evaluate defects with various depth sizes. The results indicated that the maximum relative error occurred when the depth size was 4 mm, reaching 7.5%. In the experimental section, three sets of defects with different embedded depths and depth sizes were evaluated. The experimental data showed that the maximum relative error was 5% when detecting a depth size of 2 mm. During the discussion, the influence of defect width and length sizes on the characteristic current was investigated. After comparative analysis, it was found that the defect width and length had minimal effects on the characteristic current. In conclusion, the double-sided scanning evaluation method by step magnetization can effectively evaluate the depth size of defects.

It is worth noting that when the characteristic current increases, the ability of the characteristic current to characterize the depth size of the defect will decrease, which makes an inaccurate evaluation of the distance between the defect boundary and the surface of the specimen. As a result, it affects the evaluation of the depth of the defect by the double-scanning method. In order to reduce the error, methods to improve the accuracy of the characteristic current will be discussed in future work.

## Figures and Tables

**Table 1:** Simulation parameter settings.

Simulation Module	AC/DC
Magnetizing coil	1000 turns.
Probe (the size parameters of the excitation coil and the detection coil are the same)	100 turns, Frequency = 100 kHz, Voltage = 1 V, Inner diameter 2.5 mm, Outer diameter 4 mm.
Specimen	$w_{1}$ = 200 mm, $l_{1}$ = 100 mm, $t_{1}$ = 20 mm.
Magnetic yoke	$w_{2}$ = 20 mm, $l_{2}$ = 50 mm, $t_{2}$ = 80 mm, d = 100 mm.

**Table 2:** Magnetization curve data of No. 45 steel.

H (A/m)	B (T)
0	0
245.8	0.089
414.6	0.185
550.5	0.287
673.6	0.402
818.6	0.571
996.4	0.748
1239.6	0.897
1723.8	1.091
2375	1.259
3078.8	1.378
4245.2	1.497
6495.1	1.632
9429.7	1.747
11,910.6	1.813
16,018.6	1.866
19,201.7	1.87

**Table 3:** Evaluation error.

Actual Size of Defect	Depth of Burial 2 mm	Depth of Burial 5 mm	Depth of Burial 10 mm	Depth of Burial 12 mm	Maximum Relative Error
2.0 mm	1.9 mm	1.9 mm	2.0 mm	2.0 mm	5%
3.0 mm	2.9 mm	2.9 mm	3.0 mm	2.9 mm	3.3%
4.0 mm	3.7 mm	4.0 mm	4.1 mm	3.9 mm	7.5%

**Table 4:** Evaluation error.

Actual Size of Defect	Depth of Burial 3 mm	Depth of Burial 9 mm	Depth of Burial 12 mm	Maximum Relative Error
2.0 mm	2.1 mm	2.0 mm	2.1 mm	5%
3.0 mm	2.9 mm	3.0 mm	3.0 mm	3.3%
4.0 mm	3.9 mm	3.9 mm	4.0 mm	2.5%
5.0 mm	5.0 mm	5.0 mm	5.0 mm	0%

## Data Availability

Data are contained within the article.
